# Successful Human Spermatogonial Stem Cells Homing in Recipient Mouse Testis after *In Vitro* Transplantation and Organ Culture

**DOI:** 10.22074/cellj.2019.5675

**Published:** 2018-08-07

**Authors:** Mahdi Mohaqiq, Mansoureh Movahedin, Zohreh Mazaheri, Naser Amirjannati

**Affiliations:** 1 Department of Anatomical Sciences, Faculty of Medical Sciences, Tarbiat Modares University, Tehran, Iran; 2 Department of Andrology and Embryology, Reproductive Biotechnology Research Center, Avicenna Research Institute, ACECR, Tehran, Iran

**Keywords:** Azoospermia, Human, Transplantation, Spermatogonia, Tissue Culture

## Abstract

**Objective:**

*In vitro* transplantation (IVT) of spermatogonial stem cells (SSCs) is one of the most recent methods in transplantation in recent decades. In this study, IVT and SSCs homing on seminiferous tubules of host testis in organ culture have been studied.

**Materials and Methods:**

In this experimental study, human SSCs were isolated and their identities were confirmed by tracking their promyelocytic leukemia zinc finger (PLZF) protein. These cells were transplanted to adult azoospermia mouse testes using two methods, namely, IVT and *in vivo* transplantation as transplantation groups, and testes without transplantation of cells were assigned in the control group. Then histomorphometric, immunohistochemical and molecular studies were done after 2 weeks.

**Results:**

After two weeks, histomorphometric studies revealed that the number of subsided spermatogonial cells (SCs) and the percentage of tubules with subsided SCs in IVT and *in vivo* groups were significantly more than those in the control group (P<0.05). Immunohistochemical studies in the transplantation groups confirmed that the PLZF protein was expressed in the cells subsided on the seminiferous tubule. Quantitative reverse-transcription polymerase chain reaction (qRT-PCR) demonstrated that the PLZF gene expression was only positive in the transplantation groups, but it was not significantly different between the IVT group and the *in vivo* group (P>0.05).

**Conclusion:**

Testicular tissue culture conditions after SSC transplantation can help these cells subside on the seminiferous tubule basement membrane.

## Introduction

Some male germ cells called spermatogonial stem cells (SSCs) exist on the basic membrane of seminiferous tubules in testis and participate in spermatogenesis (1). These SSCs initiate a process through which genetic information is transmitted from parents to offspring (2). Seven percent of the male population affects infertility and 10% of infertile men are azoospermic (3), so the induction and resumption of spermatogenesis with SSCs to produce mature and active sperms are among the important goals of reproductive medicine, and these goals can be achieved through SSC transplantation.

Germ cell transplantation technology has also provided new perspectives in the analysis of living environment of stem cells and in the assessment of their functions to determine their actual characteristics (4-6). SSCs can be transplanted to a recipient’s testes via two modes, namely, transplantation under *in vivo* and *in vitro* conditions. In vivo transplantation has been extensively investigated and successful results have been obtained. Other researchers examined SSC transplantation in different species (7-10).

However, this issue is even more important in cancer patients who are exposed to chemotherapy and radiotherapy treatments because of the high risk of returning cells to cancer patients prior to treatment (11). In other hand, the exclusion of malignant cells from germ cells is a big challenge. Development of a procedure to isolate testicular germ cells from malignant cells and to avoid contamination is in progress (12, 13). It is too safe that these cells after elimination of malignant cells use to *in vitro* transplantation (IVT) to host testes. For these reasons, this procedure must be conducted *in vitro*, and rigorous follow-up sessions are required. Another limitation of *in vivo* transplantation is the immune system reaction of a recipient. So, it is resulted that IVT is a good choice for these situation in we cannot transplant *in vivo*. 

Therefore, Sato et al. (14) proposed IVT in Japan for the first time. In IVT, mouse SSCs are transplanted to the testes of an azoospermia prepuberty mouse and the testes of the recipient are subsequently cultured under tissue culture conditions. As a result, sperms are produced. When SSCs are transplanted into the seminiferous tubules of azoospermia testes, SSCs migrate into the niche, where they induct and maintain a new spermatogenesis. While it is well known that primordial germ cells (PGCs) migrate into genital ridges during embryonic development, the transplantation of SSCs now demonstrates that postnatal SSCs retain the ability to migrate into their niche (15). Despite the success of IVT and tissue culture systems, studies on the IVT of human SSCs homing to adult mouse testes have not yet been conducted. 

Considering the importance of achieving *in vitro* spermatogenesis and establishing a tissue culture environment, we aimed to develop a suitable *in vitro* recipient testis for the homing and resumption of spermatogenesis with human SSCs.

## Materials and Methods

### Isolation and culture of human spermatogonial stem cells 

In this experimental study, samples were obtained from five patients with obstructive azoospermia after therapeutic testicular sperm extraction (TESE) was completed, and the remaining samples were collected after informed consent was acquired. The testis tissue were washed with phosphate buffer serum (PBS, Invitrogen, UK) and subjected to two-step enzymatic digestion according to the technique suggested by Mirzapour et al. (16) with trypsin (0.5 mg/ml, Sigma, USA), collagenase (0.5 mg/ml, Sigma, USA) and DNase (0.05 mg/ml, Sigma, USA) enzymes. Because of the low initial number and purity of SSCs in the TESE biopsy after enzymatic, and elimination of other cell types such as blood cells and so one, these cells were cultured in a testicular cell suspension for two weeks in Dulbecco’s minimum essential medium (DMEM, Gibco, UK). The number of spermatogonial cells was counted using a hemocytometer, and cell viability was determined with trypan blue.

### Immunocytochemistry identification of spermatogonial stem cells

The identity of isolated and purified SSCs was verified by tracking the PLZF protein (17) in the obtained colonies from the cell suspension. PLZF protein, as a marker of stem cells, was detected in the SSC-derived colonies through immunocytochemistry on day 7 of culturing. In brief, the cells grown on glass slides were fixed for 20 minutes in 4% paraformaldehyde at room temperature and then rinsed with PBS. The cells were permeabilized with 0.2% Triton X-100 (MP Biomedicals, USA) for 1 hour to facilitate antibody penetration, and the slides were washed with PBS supplemented with 0.2% bovine serum albumin (Vector Laboratories, USA). Nonspecific antigens were blocked with 10% normal goat serum (Vector Laboratories, USA), and the slides were then incubated overnight at 37°C with a mouse monoclonal anti-human PLZF antibody (diluted 1:100, Santa Cruz Biotechnology, USA). The slides were washed with PBS and secondary antibody (goat Texas red-conjugated anti-mouse IgM, diluted 1:100, Sigma, USA) was applied for 2 hours at room temperature in the dark.

### Preparing agarose support layer for tissue culture

To set up an agarose support layer and a culture medium with specific compositions and growth factors, we used the method described by Yokonishi et al. (18). In particular, 1.5% agarose (Carl Roth, Germany) solution was prepared and sterilized. Segments with dimensions of 1 cm×1 cm×0.5 cm were arranged by scalp considering sterile condition. They were then placed in a six-well Petri dish containing alpha minimum essential medium (aMEM, Bio-Ideal, Iran) comprising 10% knockout serum replacement (KSR), 60 ng/ml progesterone (Invitrogen, UK), 30 ng/ml beta-estradiol (Pepro Tech, USA), 20 ng/ ml epithelial growth factor (EGF, Pepro Tech, USA), 10 ng/ml human basic fibroblast growth factor (bFGF, Pepro Tech, USA), 10 ng/ml human glial cell line-derived neurotropic factor (GDNF, Pepro Tech, USA) and 10 ng/ml leukemia inhibitory factor (LIF, Royan, Iran). Pieces of recipient testicular tissues were placed gently in the middle of the agarose layer after transplantation to prevent them from floating. The culture medium was replaced twice a week.

### Labeling and *in vitro* transplantation of spermatogonial stem cells to recipient testes

**Fig.1:**
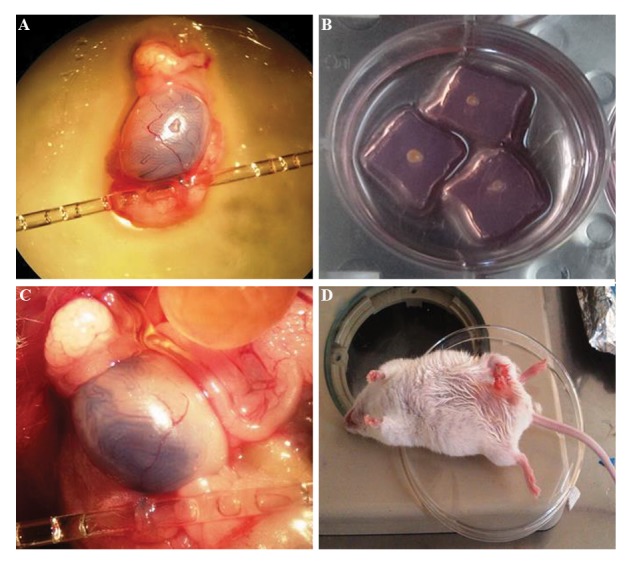
Transplantation procedure was done in host testes in different groups. **A**. *In vitro* transplantations of spermatogonial stem cells (SSCs) to azoospermia host mouse testis, **B**. Testicular tissue cut to small pieces after *in vitro* transplantation and they were placed on agarose gel after transplantation, **C**. *In vivo* transplantations of SSCs to azoospermia host mouse testis, and **D**. Preservation of host testis in mouse body.

To track the transplanted cells and distinguish them from testicular endogenous cells, we stained the cultured human SCs with Dil (2 µg/ml, Eugene.OR, USA) at room temperature and incubated them at 4°C in the dark for 20 minutes (19). Staining of the cells was verified under a fluorescent microscope, and the cells were washed with PBS. They were then isolated from a Petri dish by using 25% trypsin enzyme in 0.04% EDTA (Sigma, USA), washed three times, and transplanted to the testis of recipient mouse. To create an azoospermic model, we treated 10 recipient mice with 40 mg/kg Busulfan drug (Sigma, USA). Upon administration of this treatment, the testes of mice have a few spermatogonial cells and sertoli cells after 4 weeks. These mice were then stored in an animal house in Tarbiat Modares University, Faculty of Medical Sciences (Tehran, Iran) under the right conditions. The SSCs were transplanted into the testes of the recipient via two methods, namely, IVT (20) and in vivo transplantation (21). The host testes in the IVT group (Fig .1A) were cut into 15 small pieces (1×1 mm3) under a stereomicroscope and used to tissue culture conditions on the agarose support layer (Fig .1B). The host testes in the *in vivo* group (Fig .1C) remained in the mouse body (Fig .1D). To conduct IVT, we transplanted the SSCs into the removed testes according to a previously described protocol (22). In this protocol, a glass needle was inserted into the efferent ductuli, and the cells were injected into the end of the efferent ductuli and the opening of the rete testes. Afterward, a 10 µl cell suspension containing 105 cells was stained with trypan blue. After transplantation was completed the cell suspension was spread in the seminiferous tubules, and approximately 40 to 80% of the testis was filled. 

### Morphometric studies

An optical microscope equipped with an ophthalmologic eye lens and image-j software was used to measure various structural parameters in the sections prepared from the testes in the groups (11, 22-24). Five sections of 5µm thickness with equal spacing were selected from each testis. After staining each section with hematoxylin and eosin (H&E), 10 rounded or close-circle seminiferous tubules were randomly selected. The following formula was used to obtain the number of K(NA) 2 germ cells per unit volume (Nv):$\mathrm{Nv}=\frac{K{\left(\overset{-}{\mathrm{NA}}\right)}^{\overset{-}{2}}}{B{\left(\overset{-}{\mathrm{Vv}}\right)}^{\frac{1}{2}}}$ , where K is the constant coefficient ranging from 1.02 to 1.1; B is the ratio of the large diameter of cell to its small diameter; NA is the number of cells per unit area, and VV is the volumetric density. NA and V_V_ were calculated by image-j software. 

Two histological sections were prepared from each recipient testis with an interval of 12 µm to obtain the percent of tubules with SSCs subsiding on the seminiferous tubules (25), and all of the sections were stained with H&E. The number of the cross-sections of the tubules with homing SSCs, described as the presence of single SCs layer in the entire circumference of the seminiferous tubule, was recorded for one section from each testis.

### Quantitative reverse-transcription polymerase chain reaction 

PLZF as a pluripotency gene in a testicular tissue fragmentwas evaluated after two weeks. Total RNA was extracted from the tissue fragments of all of groups on day 14 by usingRNX-PlusTM (Cinnagen, Iran) according to the manufacturer’srecommendations. RNA concentration was then determined using a UV spectrophotometer (DPI-l, Qiagen, Iran). cDNAwas synthesized from 1000 ng RNAwith a Revert AidTM first- strand cDNAsynthesis kit (Fermentase, Lithuania) using oligo(dT) primers. PCRs were performed using Master Mix andCYBER Green I (Fluka, Switzerland) in Applied BiosystemsStepOneTM instrument (Applied Biosystems, USA). The PCR program was started with an initial melting cycle at 94°C for 4 minutes to activate the polymerase and followed by 40cycles of a melting step (20 seconds at 94°C), an annealingstep (30 seconds at 57°C), and an extension step (20 secondsat 72°C). After the PCR run was completed, the quality of thereactions was confirmed through melting curve analyses. Foreach sample, the reference gene (*ß-actin*) and the target genewere amplified in the same run. Comparative CT method (2^-ΔΔCT^) was used to determine the relative quantification ofthe target gene normalized to a housekeeping gene (*ß-actin*). The primer sequences of *PLZF* gene is:

F: 5'-GTACCTCTACCTGTGCTATGTG-3'
R: 5'-TGTCATAGTCCTTCCTTCATCTC-3'
*ß-actin* is:
F: 5.-TCCCTGGAGAAGAGCTACG-3'
R: 5.-GTAGTTTCGTGGATGCCACA-3'.

### Statistical analysis

One-way ANOVA and Tukey’s post tests were conductedto determine the statistical significance of the observeddifferences in the mean of experimental groups by usingthe SPSS statistical software (SPSS 16.0 production modefacility, SPSS Inc, USA). Data are presented as mean ± SE. Each data point represented the average of three separateexperiments, and five repeats were set up in each experiment. P<0.05 indicated statistical significance. 

### Ethical consideration 

The experimental stages in this research were in accordancewith the approval of the Ethics Committee of Tarbiat Modares University (Approval No. IR.TMU.REC.1394.68).

## Results

### Expression of the PLZFprotein to confirm spermatogonial stem cells identification

**Fig.2:**
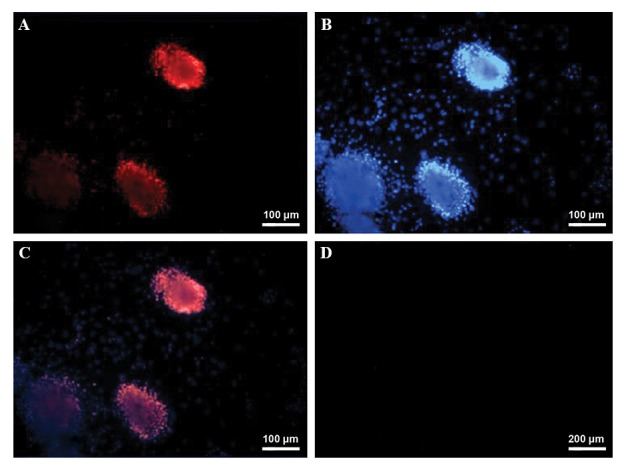
Detection of PLZF positive cells, using immunoflorescent staining, in spermatogonial stem cells (SSCs) derived colonies. **A**. Red florescent cells are PLZF positive in the obtained colonies, observing under immunoflorescent microscope, **B**. All of cells were stained with DAPI, **C**. Merge of (A) and (B), and **D**. Negative control group. These cells were observed under immunofluorescence microscope.

The cell suspension, containing SCs and Sertoli cells, was obtained and placed under culture conditions. Immunocytochemistry revealed that the colonies derived from the SCs, including SSCs, expressed the PLZF protein (Fig .2). 

### Morphometric studies

**Fig.3:**
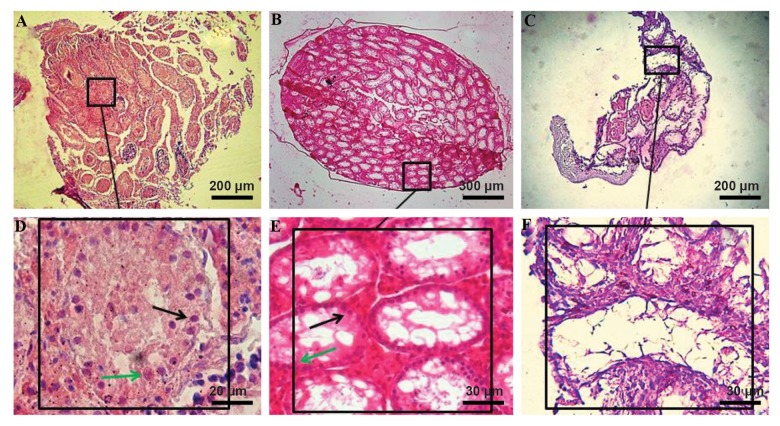
Hematoxylin-eosin staining of the testicular sections after two weeks of organ culture. **A**. *In vitro* transplantation group, **B**. *in vivo* transplantation group, **C**. Control group without transplantation, and **D, E , F**. High magnification of host testes (black arrow; SCs and green arrow; Sertoli cells).

**Fig.4:**
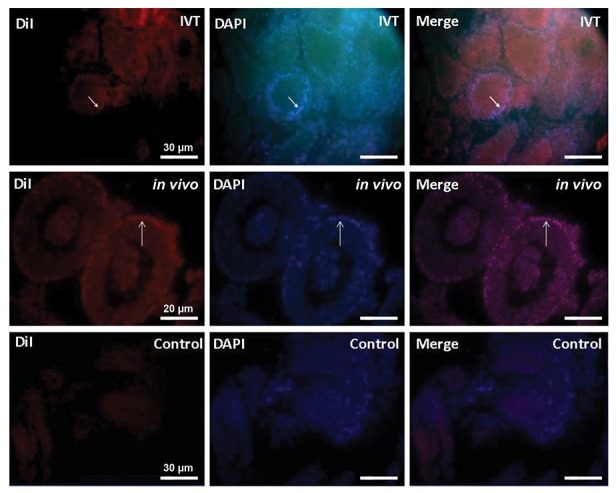
Tracing DiI two weeks after transplantation in different groups, observing under immunofluorescent microscope. Cells on the seminiferous tubules are DiI positive In *In vitro* transplantation (IVT) and *in vivo* group and DiI negative in control group. DAPI staining was also done to show all cells. DiI positive cells are human nature origin, while they after two weeks culture were transplanted to host testes (white arrow shows the DiI positive SSCs).

**Fig.5:**
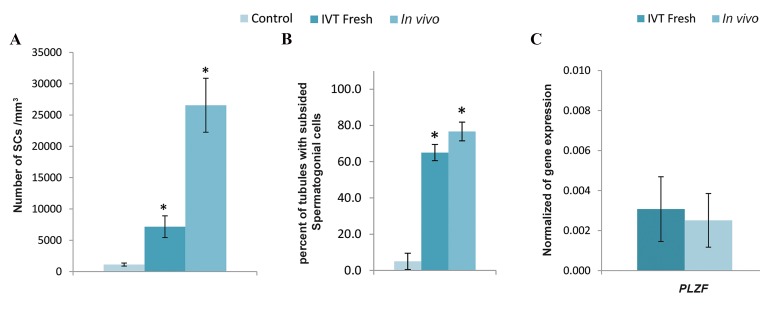
Morphometric and molecular studies in different groups. **A**. Number of SCs subsided on seminiferous tubules in different groups two weeks after transplantation, **B**. Percent of seminiferous tubules with SC homing in different groups, two weeks after transplantation, and **C**. Gene expression of PLZF in different groups two week after transplantation, normalized to *ß-actin* gene as internal control. The PLZF gene expression in control group was undetected. ^*^; significant difference compared to the other groups (P<0.05).

Our results in H&E staining studies showed that transplanted cells were deposited on the basement membrane of the seminiferous tubules in the IVT and *in vivo* groups (Fig .3). Dil tracking revealed that majority of the cells in the transplantation groups were Dil positive (Fig .4). Morphometric studies indicated that the number of SCs subsided on the seminiferous tubules in the transplantation groups were significantly more than that in the control group (P<0.05, Fig .5A). The average number of the subsided SCs in the IVT, *in vivo* and control groups were 7171.31 ± 1734.68 per mm3, 26559.7 ± 4310.37 per mm3, and 1225.67 ± 238.01 per mm3, respectively. Furthermore, the percentage of tubules with SC homing in the *in vivo* group was significantly more than that in the IVT and control groups (P<0.05). The percent of tubules with SC homing in the IVT group was significantly higher than that in the control group (P<0.05, Fig .5B). The averages of percentage for tubules with SC homing were 65 ± 4.5%, 80 ± 8.9%, and 6.7 ± 5.2%.

### Molecular analysis of PLZF gene expression and immunohistochemistry studies

The results demonstrated that the human PLZF expression was positive but was no significantlydifferent (P>0.05) in the IVT and *in vivo* groups onday 14. Human PLZF expression in the control groupwithout the transplanted human SCs was undetected, so it was not shown in (Fig .5C). 

**Fig.6:**
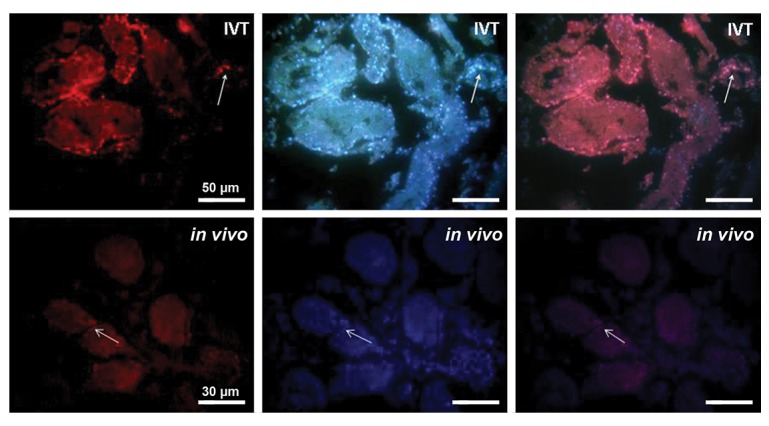
Immunohistochemistry analysis of PLZF protein in the sections of transplantation groups after two weeks. Positive PLZF expression in the spermatogonial cells on the basement membrane of seminiferous tubules In *In vitro* transplantation (IVT) and *in vivo* group. DAPI staining of whole cells were also done to show whole of cells. White arrow shows the PLZF positive SSCs.

To confirm the nature of cells subsided on the basement membrane of seminiferous tubules, we detected PLZF protein in these cells, and the results revealed that this protein was positively expressed in the transplantation groups (Fig .6). 

## Discussion

Autologous germ cell transplantation is a potential approach to restore fertility, especially for childhood cancer survivors who have become infertile due to cytotoxic therapies to treat cancer (26). At the first, elimination of potential contamination of donor germ cells with malignant cells is necessary, in advance to consider germ cell transplantation as a safe option (27). On the other hand, testis tissue culture can provide a safe system to induce spermatogenesis out of host body. So, it is resulted that transplantation and organ culture of host testis can resolve this problem. Consistent with our results observed by Mirzapour et al. (21), the recipient’s testicular tissues in the *in vivo* group supported homing of transplanted cells. They transplanted human SSCs into the mouse testis and found that these cells adhere to the basement membrane of seminiferous tubules after 2 weeks *in vivo* condition.

Our research emphasized that testicular tissue culture system could support homing of transplanted cells in the testes of recipients. Our observations in the IVT and tissue culture of the recipient’s testis were consistent with those of Sato et al. (14), who were transplanted to neonate mouse SSCs *in vitro* to an immature azoospermic testis. As a result, transplanted cells were subsided on the basement membrane of seminiferous tubules after 7-14 days. They further labeled these cells with Acrosin Green Florescent Protein (GFP) to track them; after 7-14 days, these cells are detected. 

A major problem in studying SSCs homing is that it is difficult to track SSCs immediately after transplantation (28). This is because the concentration of SSCs is very low in the testes cell suspension, and no SSC-specific markers have been identified (29). During the first several weeks after transplantation, germ cell colonies cannot be defined because of SSCs slowly proliferation (25). Kanatsu- Shinohara et al. (25) suggested strongly that B1-intergin is involved in the first several weeks of SSC colonization. Firstly, they detected a homing defect in immature pup recipient testis, demonstrating lack of the blood-testisbarrier (BTB). Because SSC homing is enhanced in pup recipients, passage through the BTB is through to be the most critical step in SSC homing (29). Testicular tissues can successfully support cells to preserve their anatomical and physical structures, because these tissues contain all cell types, including interstitial cells (30). This phenomenon is a basic requirement that provides normal conditions supporting the homing of transplanted cells on the basement membrane of seminiferous tubules. 

Our results are also consistent with those of Illien- Jünger et al. (31), who transplanted mesenchymal stem cells to an atrophied intervertebral disc exposed to complete tissue culture conditions. They observed homing of mesenchymal stem cells after 14 days and assumed that an atrophied disc tissue plays chemoattractive activities for mesenchymal stem cells. This absorptive role is probably due to secreted proteins and materials presented in a recipient’s transplanted cells. All of the cells in seminiferous tubules and interstitial tissue promote the secretion of proteins and materials that induce the absorption of transplanted SSCs existing in host seminiferous tubules. These cells then subside on the basement membrane of the seminiferous tubules. Similar to other cells, SSCs possess different membrane proteins, such as integrins (32). Integrin, as a protein in the cell membrane, plays different roles. In germline cells, PGCs, precursor of SSCs, require B1-integrin to migrate into the genital ridges during fetal development (33). For example, Potocnik et al. (34) concluded that B1-integrin protein, as an adhesion receptor, is essential for the homing of hematopoietic stem cells and they showed that these cells with a deficiency of B1-integrin of para-aortic splancno pluric are incapable of homing on embryonic and adult hematopoietic tissues. They also demonstrated that absence of integrins in hematopoietic stem cells minimizes adhesion to endothelial cells.

According to similar and confirmative results of other researchers, a testicular culture system can support not only the homing of SSCs on the basement membrane of seminiferous tubules, but also the resumption of spermatogenesis because of the secretion of proteins and materials from interstitial tissues as well as presence of the receptors and proteins on the membrane of SSCs (24). 

## Conclusion

Our results indicated that human SSCs were successfully transplanted in azoospermic mouse testis *in vitro* and homing of these cells in the testis was supported by tissue culture conditions. So, IVT and testis tissue culture system can be a good alternative to *in vivo* SSCs transplantation. It seems to be possible with this system to indicate that the *in vitro* conditions can be set up in a manner similar to the conditions in the body, so that we can do many goals that cannot be done within the body. Further studies should be performed to assay spermatogenesis after accomplishment of IVT and testis tissue culture. 
